# Robot-assisted resection of choledochal cyst in neonates

**DOI:** 10.1186/s12887-024-04942-5

**Published:** 2024-07-25

**Authors:** Sai Chen, Zhigang Gao, Qingjiang Chen, Yunzhong Qian

**Affiliations:** Department of General Surgery, National Clinical Research Center for Child Health, Children’s Hospital, Zhejiang University School of Medicine, 3333binshen Street, binjiang District, Hangzhou, Zhejiang province China

**Keywords:** Hepatojejunostomy, Neonate, Robotic, Choledochal cyst

## Abstract

**Objective:**

Laparoscopic choledochectomy and hepatojejunostomy have been reported in children since 1995, but this procedure is technically demanding. Robotic surgical systems can simplify complex minimally invasive procedures. Currently, few reports have been made on neonates. We present the experience of 6 cases of neonatal CC(choledochal cysts).

**Study design:**

Between January 2022 and December 2023, 6 neonates underwent robotic resection of choledochal cyst and hepaticojejunostomy using the Da Vinci surgical system at Children’s Hospital, Zhejiang University School of Medicine, a high-volume hepatobiliary disease center. demographic data of the patients and surgical outcomes were collected and analyzed.

**Results:**

All 6 patients were successfully treated by robotic cystectomy and hepaticojejunostomy. The mean age was 17.3 days (range 4–25) and the mean weight was 3.6 kg (range 2.55–4.4). 5 cysts were type Ia and 1 was type Iva. The mean diameter of the cysts was 3.8 cm (range 1.25-5). The mean time to establish feeding was 4.83 days (range 4–6), and patients were discharged after a median time of 16.83 days (range 7–42) without postoperative complications.

**Conclusions:**

This procedure is safe and effective for neonates. The authors found that the use of robot-assisted surgery has ergonomic advantages in this delicate, minimally invasive procedure.

## Background/purpose

The traditional management of CC has been cyst resection and Roux-en-Y hepatenterostomy. In 1995, laparoscopic-assisted surgery was first reported in children [1], but it was difficult for some surgeons to accept because of its high technical difficulty and long learning curve [2–4]. In recent years, more and more reports have suggested that robotic surgical systems can simplify complex minimally invasive surgery [5]. However, few articles have been published to describe CC in neonates, and we present our initial experience with robotic choledochal cyst resection/hepaticojejunostomy in neonates.

## Patients and methods

Between January 2022 and December 2023, 6 neonatal patients with CC were treated at our hospital. All patients underwent robot-assisted choledochal cyst resection and hepaticojejunostomy using Da Vinci XI system. All patients underwent Ultrasound and MRCP(magnetic resonance cholangiography) before operation. Informed consent was obtained from the parents of each patient.

This program was certified and approved by the Medical Ethics Committee of the Children’s Hospital, Zhejiang University School of Medicine(2024-IRB-0012-P-01).

Before the introduction of robot-assisted surgery in our hospital in 2020, we had to obtain approval from the National Health Commission. The administration of our hospital accepts the use of this new method provided that data are collected prospectively for each case and the results are regularly audited.

### Surgical procedure

#### Port placement in RACH

**Fig. 1:**
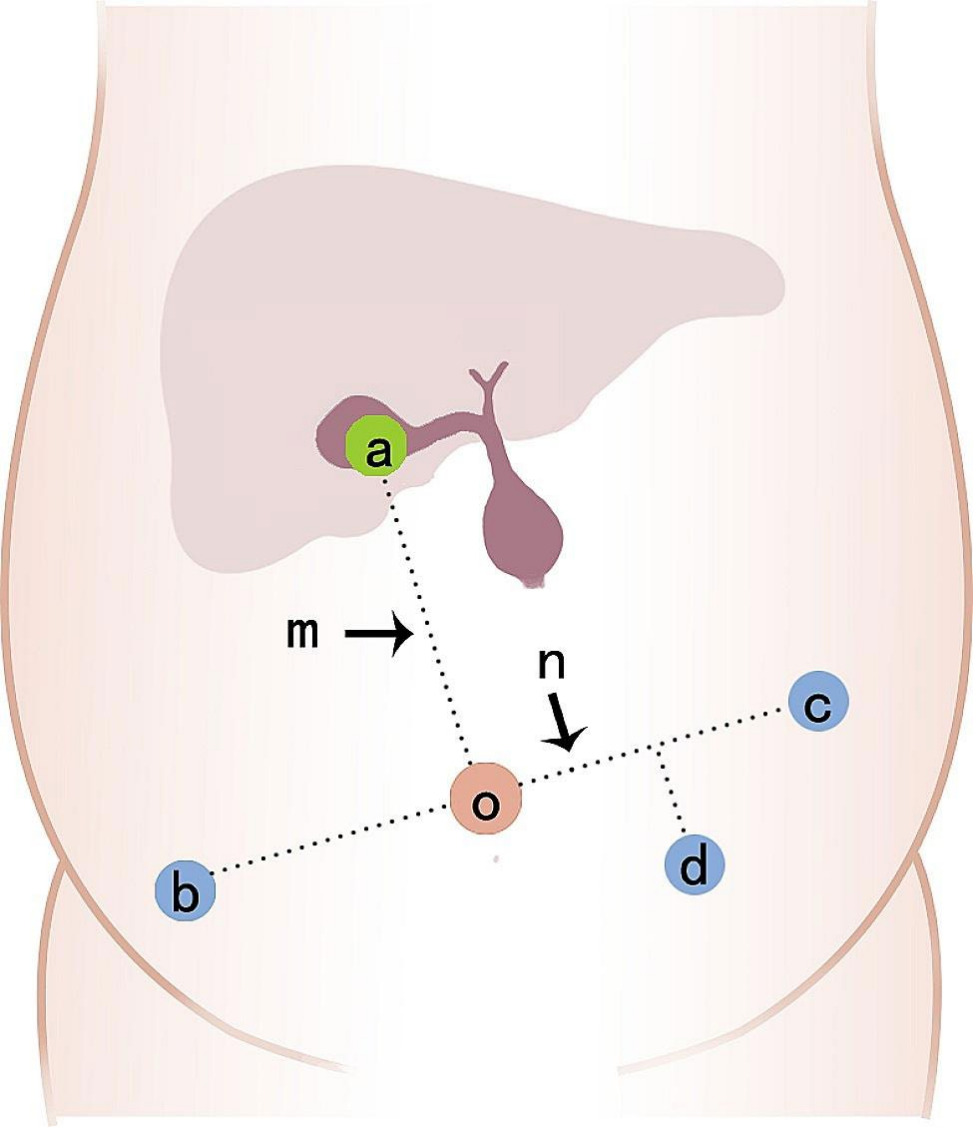
Port placement in RACH: A line (m) was drawn from the umbilicus to the right upper abdomen (projection of the gallbladder floor surface, (**a**) as two points, and a line (n) was drawn perpendicular to this line through the umbilicus (o). Two points (numbers **b** and **c**) were taken on the n line about 4 cm from the umbilicus. Take the perpendicular line between (**b** and **c**), and take a point about 3 cm below (**d**). Numbers b, o, and c are all Da Vinci trocar placement positions (8 mm), and number (**d**) is the assistant surgeon’s operating port (5 mm)

Endotracheal intubation was performed under general anesthesia. The patient was placed in a supine position and the operating table is tilted to 0–15° (head height low) as appropriate. Pneumoperitoneum was established according to routine procedure, and the pressure was set at 6 mmHg (1 mmHg = 0.133 kPa). An opening was made for the laparoscope in the midumbilicus using an 8-mm Trocar, which served as the port for the 3D camera (o number). Two additional 8 mm trocar were placed in the left upper abdomen (number b) and right lower abdomen (number c) approximately 3 cm from the umbilical incision, and a 5 mm laparoscopic trocar was placed in the left abdomen (d) to be used by the assistant surgeon as an auxiliary oral (Fig. 1).

#### Steps of choledochal cyst resection and Roux-en-Y hepaticojejunostomy in RACH

**Fig. 2:**
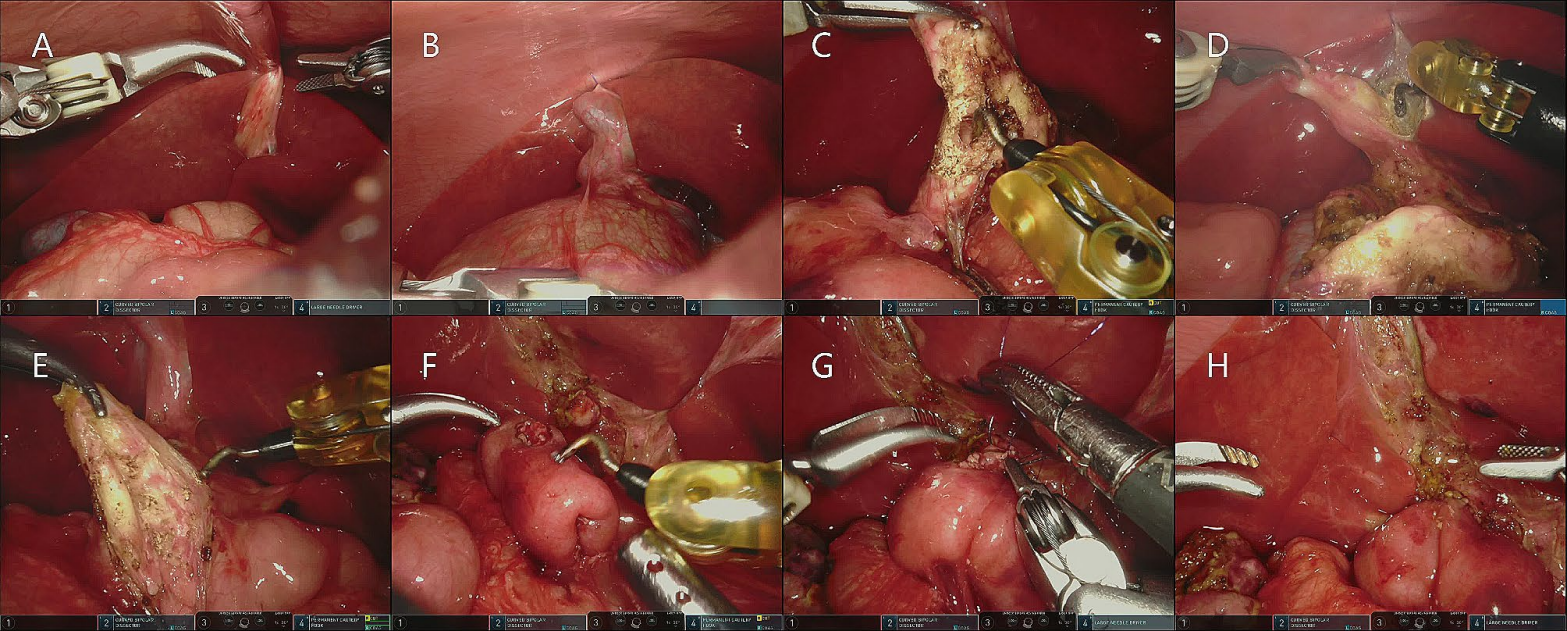
Steps of choledochal cyst resection and Roux-en-Y hepaticojejunostomy in RACH: (**A**) Suspension of the round hepatic ligament (**B**) Suspension of the gallbladder (**C**) Decompression of the common bile duct (**D**) Cholecystectomy (**E**) Resection of the cyst to the common hepatic duct (**F**) Reserving the common hepatic duct and jejunum (biliary branch) (**G**) Suturing the common hepatic duct and jejunum continuously (**H**) Evaluating the tightness of CJ reconstruction according to the deformation of the hepatic duct and jejunum Visually

The round ligament of the liver (Fig. 2A) and the gallbladder (Fig. 2B) were suspended with 3 − 0 absorbable sutures and pulled upward to fully expose the hepatic hilum. A transverse incision was made on the anterior wall of the cyst below the cystic duct opening and decompression was performed to detect the vagal bile duct opening, as shown in Fig. 2C. The gallbladder was gradually detached from the base to the neck using an ultrasonic scalper or electrocoagulation hook, and the cystic artery was ligated to expose the biliary cyst, as shown in Fig. 2D. The distal end of the cyst wall was lifted upward, and the cyst wall was gradually dissociated from the proximal end to the distal end, exposing the distal end of the common bile duct until it was close to the thinner part of the pancreatic duct. Due to the thin bile duct in neonates, it was generally not ligated. Next, the cyst at the proximal end of the common bile duct was cut, dissociated upward to the common hepatic duct, and the remaining cyst wall was removed together with the gallbladder, as shown in Fig. 2E.

The opening of the common hepatic duct was trimmed for use. The jejunum was secured with a pair of intestinal forceps 15 to 20 cm from Treitz’s ligament and pulled out of the abdominal cavity through a transumbilical incision. The appropriate length of the jejunal loop was determined based on the straight distance between the umbilicus and the hepatic hilum. The proximal jejunal loop was closed, and the distal jejunal loop was anastomosed to the proximal jejunum. The jejunum was inserted into the abdominal cavity, and a tunnel was created in the right mesocolic avascular zone of the middle transverse colic artery through which the jejunal loop was elevated to the hepatic hilum. The intestinal wall was cut 1 cm from the blind end of the jejunal loop, the length was the same as the size of the opening of the common hepatic duct, and a biliary branch was formed for reserve. As shown in Fig. 2F.

The common hepatic duct and jejunum (biliary branch) were sutured with a full-thickness continuous suture using a PDS-II needle, as shown in Fig. 2G. After the closure, the anastomosis was checked again to visually evaluate the tightness of CJ reconstruction based on the deformation of the hepatic duct and jejunum. As shown in Fig. 2H. The abdominal cavity was flushed, a drainage tube was placed behind the anastomosis, and the external body was extracted through the right puncture hole. The trocar was removed and the incision was sutured.

All patients who underwent choledochal cyst resection in our department underwent abdominal ultrasound scan, blood routine and liver function tests at clinical follow-up of 2 weeks, 4 weeks, 3 months, 6 months and annually thereafter. To date, patients have been followed for an average of 7.97 months (1.2–19.9 months). All data were collected prospectively.

## Results

Robotic choledochal cyst resection and hepaticojejunostomy were successfully performed in 6 neonates, as shown in Table 1. Only 1 very low birth weight infant was born at 28 weeks of age. All the other patients were full-term normal birth weight. 5 patients were female and 1 was male, with a mean age of 17.3 days (range 4–25) and a mean weight of 3.6 kg (range 2.55–4.4). 4 cases were detected prenatally, 1 with jaundice and another 1 with abdominal mass.

**Table 1:** Patient characteristics

NO.	Gender	Age(Gestational), WK ^a^	Age ^b^(Operation), D	Weight(Birth), Kg	Weight(Operation), Kg	Chief complaint	Comorbidity
1	Female	39	22	3.8	4.4	Prenatal examination	
2	Male	41	25	4.1	4.3	Jaundice	Bilateral indirect inguinal hernia.
3	Female	39	19	3.2	3.7	Prenatal examination	
4	Female	28	15	1.2	2.6	Abdominal mass	VLBW ^c^, premature infant, pneumonia.
5	Female	40	4	3.4	2.7	Prenatal examination	
6	Female	38	19	3.3	4	Prenatal examination	

*WK ^a^: week, Age ^b^After correcting for gestational age, VLBW ^c^very low weight baby

The intraoperative findings in Table 2 show that only 1 of the 6 children was type IVa, the rest were type Ia, with cysts ranging in diameter from 1.25 cm to 5 cm, and 5 cases were accompanied by stones. The average diameter of anastomosis was 1.13 cm. The average robot operation time was 202.83 min (range 154–340).

The postoperative situation in Table 3 indicates that the 6 children started feeding at a median of 4.83 days (range 4–6), and the children were discharged from the hospital a median of 16.83 days (range 7–42) after surgery.

**Table 2:** Operative outcomes

NO.	Todani classification	Diameter of cyst, cm	Calculi	Diameter of anastomosis, cm	Duration of surgery, min	Intraoperative bleeding, ml
1	Ia	5	1	1	165	2
2	IVa	3.5	1	1	180	5
3	Ia	4.6	1	1.5	206	2
4	1a	4.5	0	0.8	340	2
5	1a	4	1	1.5	154	2
6	1a	1.3	1	1	172	5

**Table 3:** Postoperation outcomes

NO.	Postoperative fasting time, D	Postoperative hospital stay, D	Cost of hospitalization, CNY	Follow-up time, Mth
1	5	18	79235.79	7.6
2	4	17	93879.69	5.8
3	6	9	79089.83	5.1
4	5	42	142751.38	8.3
5	5	8	90279.4	20.7
6	4	7	87220.68	23.8

There was 1 premature infant with a birth weight of only 1.17 kg, which was a very low birth weight infant. At the time of surgery, the child was 15 days old (After correcting for gestational age)and his weight increased to 2.55 kg. The operation was successful. However, the children have more severe pulmonary infection, which leads to longer postoperative hospital stay and higher postoperative costs.

Each child underwent a postoperative follow-up, during which ultrasound images and blood results were normal, and the parents did not report any complications.

In Table 4, we compared these 6 neonates who underwent RACH with 11 neonates who underwent LAHJ during the same period and found that children in the RAHJ group had higher costs and less intraoperative bleeding, *p* < 0.05. There was no significant difference in other results between the two groups.

**Table 4:** Statistical tables of neonatal data for two surgical procedures

		RAHJ^a^	LAHJ^b^	*p* Value
No. procedures N		6	11	
No. Gender:				
	Male N(%)	1 (16.7)	1 (9.1)	
	Famale N(%)	5 (83.3)	10(90.1)	
No. Abnormalities:				
	Prenatal examination N(%)	4 (66.6)	6(54.5)	
	Abdominal mass N(%)	1 (16.7)	1(9.1)	
	Jaundice N(%)	1(16.7)	4(36.3)	
Median Age^c^ (IQR), d		17.3(12.3, 22.8)	14.9 (7, 26)	0.525
Median Bodyweight(IQR), kg		3.9(2.7–4.3)	3.7(3.1, 4.5)	0.961
Median Duration of surgery(IQR), min		202.8(162.3, 239.5)	180.2(162, 216)	≥ 0.99
Median Intraoperative blood loss(IQR), ml		3 (2, 3)	5.9 (5, 5)	0.048
Median Postoperative fasting time(IQR), d		4.8(4, 5.3)	7.1 (4, 8)	0.525
Median Postoperative time of Stay(IQR), d		16.8(7.8, 24)	15.4 (10, 28)	0.591
Median Practice expense (IQR), CNY		95409.5(79199.3, 106097.6)	41995.5(32891.9, 53,463)	<0.001
Median Follow-up time (IQR), Mth		11.9(5.6–21.5)	46.2(30.7–59.2)	<0.001
Biliary fistula N(%)		0(0)	1(9.1)	≥ 0.99

*RAHJ^a^: Robot-assisted hepaticojejunostomy; LAHJ^b^, Laparoscopic-assisted hepaticojejunostomy

Age^c^, After correcting for gestational age

## Discussion

Since Woo first performed the RAHJ procedure in a child in 2006 [6], the safety of robotic surgery in the treatment of CCs in children has been reported. However, subsequent studies by Dawrant., Meehan, and Alizai et al. have shown that robot-assisted surgery has advantages in the application of complex hepatobiliary surgery [7–9]. Moreover, compared with adults, children’s tissues and organs are smaller and more fragile, and the space for surgery is smaller, requiring higher accuracy and precision. So theoretically, pediatric surgery is more suitable for robotic surgery, but the current robotic surgical system is based on adults and has limited application in pediatric surgery. To the best of our knowledge, there are few reports in the literature on neonatal choledochal cysts.

In our medical center, laparoscopic-assisted choledochal cyst resection has been performed for the treatment of CC in children since 2020. In this article, we report 6 neonates, the youngest being only 4 days old, which is a smaller population than has been reported in the literature, and we did not convert any of the patients to open surgery.

Given the small intra-abdominal space of newborn infants, we take various steps to maximize this working space. The body of the newborn is so short that the operating arm of hole b (Fig. 1) is prone to infection with the right lower limb during the operation. In order to reduce the possibility of interference, the position can be adjusted as appropriate during the operation, and the operating table can be tilted to 0–15° (head high and foot low). For neonates, suspension of gallbladder and round ligament of liver is the key, which can fully expose the location of the lesion and optimize the visual field. In addition, decompression of the cyst is also very important, which can not only reduce the volume of the cyst to increase the operating space, but also expose the posterior vessels and aberrant bile ducts to avoid unnecessary damage. Finally, the distance between adjacent operating holes was shortened and the depth of Da Vinci trocar entry was reduced to maximize the range of operating space and visual space. For newborns, we would prefer to use a 5 mm or even a smaller 3mm1 trocar to reduce the damage to newborns, but unfortunately, we only have 8 mm trocars in our hospital. It is believed that with the development of technology, these shortcomings will be gradually corrected. In the future, the emergence of small robotic surgical systems and instruments, or even robotic surgical systems and instruments designed specifically for neonates, will undoubtedly make a breakthrough in the application of neonatal surgery.

Unlike older children and adults, neonatal choledochal cysts are recommended to decompressive choledochal cysts before cholecystectomy due to the limited operating space, as shown in Fig. 2. This can fully expose the gallbladder triangle and surrounding blood vessels and reduce unnecessary bleeding during operation.

Our total operative time of approximately 202 min appears to be shorter than that of patients published in the literature [8, 10]. As a national regional center, our hospital has performed a large number of conventional laparoscopic-assisted choledochal cyst resection and robot-assisted choledochal cyst resection in older children in recent years. With the experience and availability of both specialized primary surgeons and robotic surgical groups, our procedure time will undoubtedly be reduced.

Robotic surgery has the advantages of excellent visualization and instrument control [11]. Robotic surgery includes 3D imaging, tremor filters, and articulated instrumentation [12]. 3D imaging allows high definition of vision and magnification up to 10 times, resulting in more accurate vision. Tremor filter and articulated instrument can make the operation have more flexible and stable instrument joints, eliminate the physiological tremor of the operator, and operate in a delicate space and ergonomic operating position [13]. As a result, Da Vinci robot-assisted surgery has become increasingly popular and the feasibility of this system has been recognized [14, 15]. Because of the lack of space and increased maneuverability of the instruments, there are few reports on robotic surgery in low weight or young children [16–18].

The mean duration of fasting in our study was 4.83 days, which is similar to what is known in the literature for older children [6]. However, the median postoperative hospital stay was longer (RACH: 16.8 days, LACH: 15.4days), which may be related to the slow recovery of gastrointestinal function in neonates. However, there are few cases and further research is needed.

Indeed, some surgeons believe that robotic surgery is not appropriate for infants [19, 20]. Unlike conventional laparoscopic surgery, robotic surgery completely lacks tactile feedback [21]. It was not possible to assess the tightness and tension of the suture from touch. Therefore, we adapted the visual-tactile feedback method from Yusheng Shi et al. ‘s report to visually assess the tightness of CJ reconstruction based on the deformation of the hepatic duct and jejunum [22].

Another obvious disadvantage of robotic surgery is the high cost [11]. In this study, the average hospitalization cost of children was 95409.46 CNY (range79089.83-142751.38), and the hospitalization cost was relatively expensive. However, the current insurance policy launched in our city and the fund funding of our hospital, the out-of-pocket part is almost equivalent to laparoscopic surgery. Therefore, if there is a better medical subsidy policy, robotic surgery will be increasingly favored and used as the standard in many hospitals.

## Conclusion

Robot-assisted choledochal cyst resection and hepaticojejunostomy is safe and effective for neonates. The authors found an ergonomic advantage to the use of robot-assisted surgery in this complex minimally invasive procedure.

## Data Availability

All data supporting the findings of this study are available from the corresponding author (Zhigang Gao) on request after appropriate ethical approval.
